# Perceptions of the Coronavirus and COVID-19 testing and vaccination in Latinx and Indigenous Mexican immigrant communities in the Eastern Coachella Valley

**DOI:** 10.1186/s12889-022-13375-7

**Published:** 2022-05-21

**Authors:** Daniel Gehlbach, Evelyn Vázquez, Gabriela Ortiz, Erica Li, Cintya Beltrán Sánchez, Sonia Rodríguez, María Pozar, Ann M. Cheney

**Affiliations:** 1 School of Medicine, University of California Riverside, 900 University Ave, Riverside, CA 92521 USA; 2 University of California Riverside, Riverside, USA; 3 Center for Health Disparities Research, University of California Riverside, Riverside, USA

**Keywords:** COVID-19, Health disparities, Immigrant health, Latinx, Community-based participatory research, Indigenous Mexicans

## Abstract

**Background:**

A novel coronavirus, SARS-CoV-2 (known as COVID-19), spread rapidly around the world, affecting all and creating an ongoing global pandemic. Across the United States, Latinx and Indigenous populations have been disproportionately affected by COVID-19 cases and death rates. An examination of the perceptions and beliefs about the spread of the virus, COVID-19 testing, and vaccination amongst racial-ethnic minority groups, specifically Latinx and Indigenous Latin American immigrant communities, is needed to alleviate the widespread disparity in new cases and deaths.

**Methods:**

This study was carried out from August 2020 to January 2021 and used community-based participatory research to engage community partners and build the capacity of community health workers (i.e., promotores de salud) and pre-medical and medical students in conducting qualitative research. The objective of the study was to examine the structural and social determinants of health on perceptions of the coronavirus, its spread, and decisions around COVID-19 testing and vaccination. Data collection included ethnography involving observations in public settings and focus groups with members of Latinx and Indigenous Mexican farm-working communities in the Eastern Coachella Valley, located in the Inland Southern California desert region. A total of seven focus groups, six in Spanish and one in Purépecha, with a total of 55 participants were conducted. Topics covered include perceptions of the coronavirus and its spread, as well as COVID-19 testing and vaccination.

**Results:**

Using theme identification techniques, the findings identify structural and social factors that underly perceptions held by Latinx and Indigenous Mexican immigrants about the virus and COVID-19, which, in turn, shape attitudes and behaviors related to COVID-19 testing and vaccination. Common themes that emerged across focus groups include misinformation, lack of trust in institutions, and insecurity around employment and residency.

**Conclusions:**

This immigrant population is structurally vulnerable to historical and present-day inequalities that put them at increased risk of COVID-19 exposure, morbidity, and mortality. Study findings indicate a significant need for interventions that decrease structural vulnerabilities by addressing issues of (dis)trust in government and public health among this population.

## Introduction

Across the United States (US), Latinx and Indigenous Latin American communities are disproportionately at risk for contracting SARS-CoV-2, a novel coronavirus that causes the COVID-19 respiratory disease [1]. Indigenous populations are 2.3 times more likely to die from COVID-19 than White populations, [2] a widening racial-ethnic disparity in mortality. When considered from a nationwide standpoint, Latinx groups have 2 to 4 times higher rates of COVID-19 than expected for their proportion of the population in 43 of the 44 jurisdictions in the US based on reported ethnic data [3]. In California, recent estimates indicate the Latinx population represents 38.9% of the total population; however, they account for 47.1% of all cases and 43.6% of all deaths [4].

The mechanisms behind these health disparities remain unclear. Recent studies reveal structural and social factors as underlying causes to COVID-19-related health disparities in racial-ethnicity minority populations. Minority groups continue to be disproportionately affected by chronic medical conditions and experience poor access to healthcare [5]. In the context of the current pandemic, long-standing structural and societal factors that shape limited healthcare access and contribute to chronic medical conditions (e.g., diabetes, obesity, asthma) among the Latinx and Indigenous Latin American population in the US have been further identified and exposed.

In the US and across the globe, the pandemic has magnified the historically rooted systemic and social factors shaping health disparities among marginalized communities of color [6, 7]. As Frank Snowden, professor of history of medicine at Yale, pointed out, “epidemic disease are not random events that afflict societies capriciously and without warning... every society produces its own specific vulnerabilities”[8]. The coronavirus has moved and continues to move along the “fault lines created by poverty and inequality”[9]. Partnered research that engages community leaders and trusted members of the community, and acknowledges the histories of inequality and mistreatment that have shaped health disparities, is needed to mitigate the impacts of the coronavirus on vulnerable communities.

### Structural and social determinants of health

Structural inequalities and social determinants of health (SDOH) place disadvantaged individuals and populations at greater risk for contagion and death [10, 11]. The public health field has traditionally focused on SDOH, highlighting how the conditions into which individuals are “born, grow, live, work, and age—are significant drivers of disease risk and susceptibility within clinical care and public health systems”[12]. Yet, as social medicine and related fields have argued for some time, structural level factors underlie the development and distribution of SDOH and have historically set up and put into motion inequities in health [12, 13]. Historical factors such as colonization, forced repatriation, land loss, and slavery render certain racial-ethnic groups more susceptible to the harmful and detrimental outcomes of the power imbalances embedded within institutions and social life [14–17]. These inequities are seen in higher rates of cardiovascular disease, diabetes, respiratory disease, hypertension, cancer, and infectious disease in marginalized communities of color [18].

In the context of COVID-19, the intersection of structural and SDOH—sociocultural economic, political, and environmental factors coupled with histories of abuse, exploitation, and oppression, increase risk for COVID-19 transmission and death [6, 19]. Studies show income inequality (e.g., low wages, essential labor), housing and neighborhood density [20], geographic location (e.g., rural vs. urban) [21, 22], xenophobic and racialized climates [23, 24], and distrust in public health and the government [25] contribute to health disparities and higher rates of infection, hospitalizations, and deaths in underserved and vulnerable communities [19].

The objective of this study was to understand how both structural and SDOH shape perceptions of the coronavirus, its spread, and decision making around COVID-19 testing and vaccination in vulnerable populations. The research examined the intersections of structural and SDOH among Latinx and Indigenous Mexican immigrant communities in the rural borderland of inland southern California.

## Methods

### Project overview

This study was carried out from August 2020 to January 2021, with focus groups conducted from November to December 2020. We used principles of community-based participatory research (CBPR), an approach that draws on the strengths of diverse partners, shares resources, and fosters shared decision making and knowledge creation [26]. Aligning with this approach, we convened a community advisory board of 10 members with representation from healthcare systems, healthcare providers, growers, community health workers, and medical and premedical students that met monthly to oversee the project, facilitate relationship building, and offer advice. The leadership team also met regularly with partnering public health officials and healthcare leaders to discuss COVID-19 testing service delivery (e.g., location of testing sites, hours of operation) and the engagement of more vulnerable communities in testing services.

The larger project included three aims: 1) support the delivery of COVID-19 testing services, 2) broadly disseminate COVID-19 public health information, and 3) conduct research on perceptions of the coronavirus and COVID-19 testing and vaccination among Latinx/Indigenous Mexicans in rural agricultural communities in Inland Southern California. As reported elsewhere, the larger project was successful in carrying out Aims 1 and 2 of the study [27]. The team’s leadership established access to routine COVID-19 testing for rural, immigrant communities partnering with a federally qualified health center and county public health to conduct 26 testing clinics providing approximately 1470 tests. Additionally, community health workers or *promotores de salud* disseminated COVID-19 related public health information via social media, at COVID-19 testing events, and in-person socially distanced community talks. These efforts resulted in 22 virtual COVID-19 community talks (Pláticas de COVID-19) livestreamed on our Facebook page @Unidoporsalud and 10 in-person COVID-19 community talks (*Pláticas en el Pueblo*).

For the purposes of this article, we focus on the findings from our third aim involving research on the coronavirus and COVID-19 perceptions. Prior to the start of data collection, we obtained ethical approval for the study from the University of California Riverside’s Institutional Review Board.

### Study setting

Our study focused on the effects of the coronavirus pandemic amongst Latinx and Indigenous Mexican immigrant communities in the rural desert region of Inland Southern California. Our research was carried out in Riverside County, an area of California in which racial-ethnic minority populations have been disproportionately impacted by the pandemic. At the time of the study, Riverside County had the second highest number of confirmed cases and deaths in the state [28, 29]. There are over 2 million Latinos in Riverside County, a majority minority population that outnumbers all other racial and ethnic groups in the region [30–32]. Most Latinos are of Mexican origin, with smaller numbers of Puerto Ricans, Salvadorans, and Guatemalans, and Indigenous Nations [33]. Latinos in this region suffer health disparities due to low income and education, limited English proficiency, and undocumented status [34–36]. Unsurprisingly, the pandemic has severely impacted this population in the region: At research inception in fall 2020, which aligned with wave two spread of the coronavirus in California, county level data indicated the Latinx population accounted for 57% of COVID-19 cases and 46% of deaths in Riverside County [28, 29, 37, 38].

Our study focused on engaging Latinx and Indigenous Mexicans in rural agricultural communities in the eastern part of the Coachella Valley in Riverside County. The Coachella Valley, a 45-mile-long valley encompassing nine cities and rural agricultural communities, is an area of particular racial-ethnic disparity. This area is home to several vulnerable communities including the unincorporated rural communities of the Eastern Coachella Valley (ECV): Mecca, North Shore, Oasis, and Thermal, home to many Latinx and Indigenous Mexican immigrants living below the poverty line and working in the nearby agricultural fields. This region is home to the Purépecha community, an Indigenous Mexican population from the state of Michoacán [34]. At the start of the pandemic this region was identified as a hotspot, with some reports indicating a COVID-19 infection rate in the ECV 5 times higher than other Coachella Valley communities [39].

During the time of our study, these unincorporated rural communities (Mecca, North Shore, Oasis, and Thermal) consistently reported the highest rates of COVID-19 cases per 1,000 residents in the Coachella Valley. For instance, in September 2020 Thermal reported > 130 cases/1,000 residents, which increased to > 250 cases/1,000 residents in January 2021. This is significantly higher than case rates in Palm Springs (also in the Coachella Valley), which reported > 20 cases/1,000 residents in September 2020 and > 50 cases/1,000 residents in January 2021 [40].

This pattern of increased total confirmed cases of COVID-19 in these ECV communities continued throughout the study period. In September 2020, Mecca had 455 cases increasing to 1079 cases in January 2021; North Shore had 136 cases increasing to 331 cases; Oasis had 333 cases increasing to 826 cases; and Thermal had 185 cases increasing to 440 cases [40]. An increase in deaths due to COVID-19 accompanied the increased cases. In September 2020, Mecca had 9 reported deaths increasing to 16 in January 2021; North Shore had 1 reported death and remained stable; Oasis had 5 reported deaths increasing slightly to 6; and Thermal had 0 reported deaths increasing to 4 deaths [40].

### Ethnographic observations

During community advisory board meetings, meetings with partners (e.g., public health, healthcare leaders), and attendance at meetings with growers we made ethnographic observations and jotted down key discussion topics [41]. Team members reflected on these observations and used this information to inform the focus group interview guide and analysis and interpretation of the data.

### Focus group eligibility and recruitment

*Promotores de salud* recruited community members into the focus groups by distributing study flyers with eligibility criteria and study contact information to individuals and families in their social networks. Eligibility criteria were met if a community member: 1) was 18 years of age or older, 2) lived in the ECV and/or farm-working community along the Salton Sea, 3) self-identified as Latino/Hispanic, Latinx and/or indigenous from Latin America, and 4) spoke Spanish and/or Purépecha. Monolingual English-speaking Latinos and monolingual speakers of an indigenous dialect other than Purépecha were excluded from participation.

### Data collection

A focus group is a group interview that allows qualitative researchers to gather collective data about a specific phenomenon of interest. This method of data collection allows participants to build on each other’s ideas [42], providing collective (rather than individual) knowledge about the structural and socio-cultural factors shaping perceptions of the coronavirus and attitudes and behaviors around COVID-19 testing and vaccination. From November to December 2020, we conducted seven virtual focus groups (of six to ten people each) to elicit information on shared structural stressors and socio-cultural factors shaping attitudes and behaviors around COVID-19 testing and vaccination. For nonprobability samples, 80% of themes can be identified within two to three focus groups and 90% within three to six focus groups [43].

*Promotores de salud* facilitated the focus groups with assistance from medical and pre-medical students. All facilitators received training on qualitative data collection and data analysis. Facilitators used a semi-structured interview guide with open-ended questions to elicit information on shared beliefs and attitudes around the virus, its spread, and COVID-19 testing and future vaccination, as well as risk-reduction behaviors such as social distancing and use of face coverings. We prompted discussion about themes emerging from our ethnographic observations and conversations with community members during public health outreach and testing events, including trust in public health officials, the government, and providers/healthcare systems, as well as strategies and tools to support those with COVID-19 and increase risk-reduction behaviors and use of COVID-19 testing services. At the end of all focus groups, participants were asked to complete a socio-demographic survey, either by using a link to a Qualtrics (online) version of the survey, or by having a team member administer the survey to them via phone.

### Data analysis

Focus groups were conducted via Zoom video conference, audio recorded, transcribed, and analyzed using template and matrix analysis, a rapid qualitative analytic technique [44–46]. This technique involved summarizing all focus group transcripts using a template organized by the key topics of the semi-structured interview guide (template analysis). Key domains included: coronavirus, its spread, and ways to reduce virus propagation; attitudes and beliefs about COVID-19 testing, barriers to testing, and resources for people testing positive; and thoughts about COVID-19 vaccines and barriers to vaccination. A matrix was then created to organize the responses from each summary template (as rows) by key domains (as columns). *Promotores* *de salud *and students participated in a 2-part training on template and matrix analysis and led data analysis with support from experts in this analytic approach. Team members read transcripts line-by-line and inserted data, including illustrative excerpts from the interviews, in the templates. Next, a matrix (focus group × domain) was created, and data from each template were inserted into the matrix. The matrix facilitated the identification of cross-case themes/patterns across the seven focus groups conducted.

**Fig. 1:**
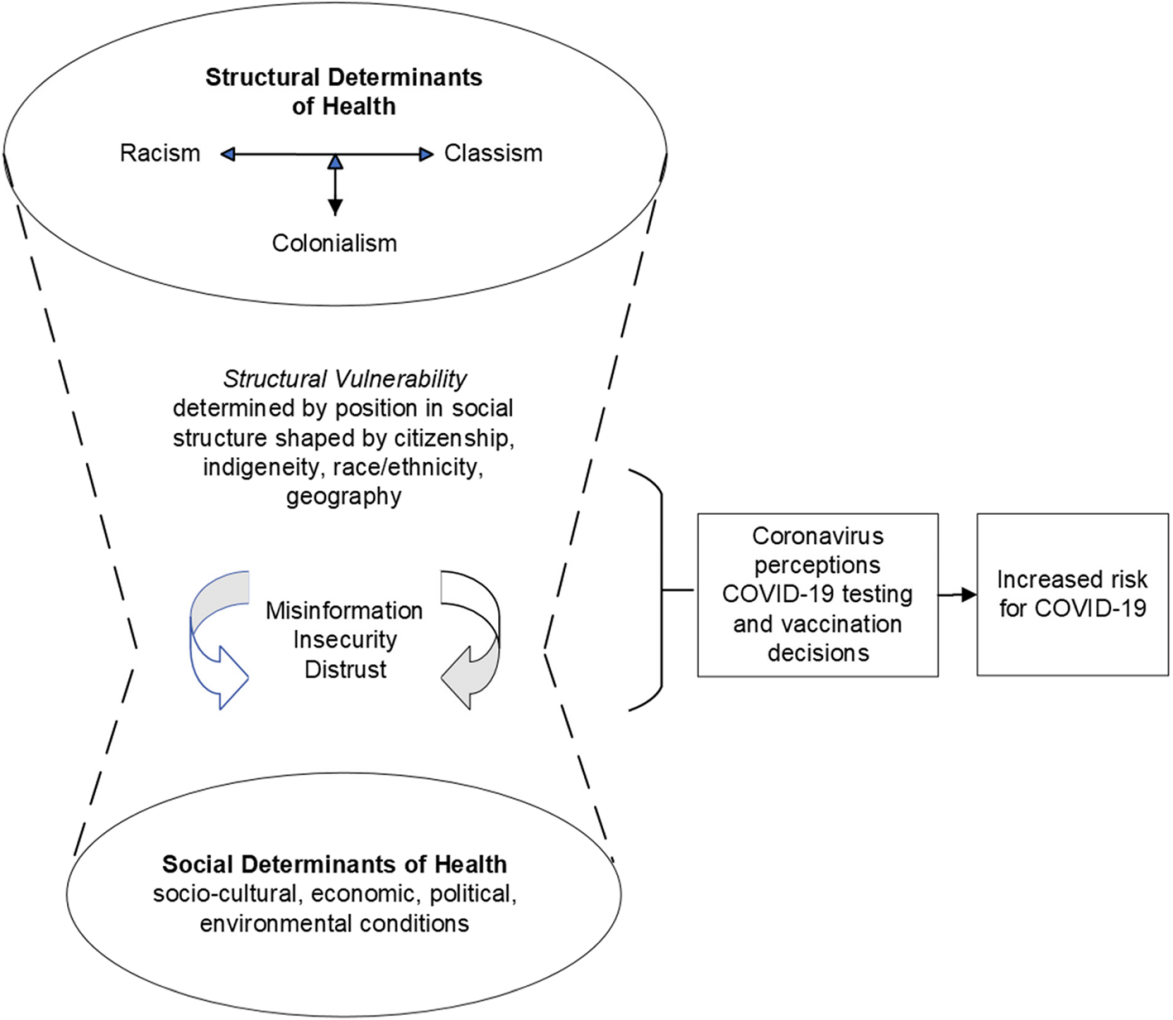
Structural and Social Determinants of Health in COVID-19 Risk

Through this iterative process of theme identification and constant comparison across cases, we developed a conceptual model (Fig. 1) grounded in the data that reflects the relationships among themes and their connection with COVID-19 testing and vaccination. We used exemplar quotes to substantiate these patterns. Participant quotes were translated from Spanish to English by the first author, a native English speaker proficient in Spanish. Graduate and PhD-level bilingual (Spanish–English) team members then checked participants quotes for accuracy and validated them.

## Results

Seven focus groups were held with 55 total participants, 53 of whom completed the socio-demographic survey (see Table 1). All participants self-identified as either Hispanic or Latino (83%) and/or Purépecha (17%). Most participants were female, age 25 to 54 (83%) and had health insurance (72%). More than half the participants represented those living in unincorporated farming communities: Thermal, Mecca, and Oasis. Over one-third (36%) of participants identified as farmworkers, suggesting a significant percent of women in the sample worked in the fields. Most (83%) participants felt affected by the coronavirus. Participants cited reduced work hours and income, inability to work or no work, childcare, and COVID-19 infection as ways they were most affected by the virus. Among survey respondents who cited childcare as a factor in how the coronavirus has affected their lives, a third theme was remote learning. The socio-demographic characteristics of study participants reflect recent population profiles of communities in the ECV regarding education, economic status, and occupation [47]. However, there is overrepresentation of immigrants, women, and individuals with healthcare insurance.

**Table 1:** Demographics and characteristics of participants

	*N* = 53
*Demographics*	N(%)
Gender	
Female	43(81)
Male	10(19)
Ethnicity/Race	
Hispanic or Latino	44(83)
Purépecha	9(17)
Age	
18 to 24	5(9)
25 to 44	21(40)
45 to 54	23(43)
55–64	4(8)
Health insurance	
Yes	38(72)
No	15(28)
Community	
Unincorporated farm-working communities (Thermal, Mecca, Oasis)	30(56)
Indio	7(13)
Salton City	3(6)
Coachella	11(21)
Other	2(4)
Employment status	
Part-time (less than 40 h/week)	18(34)
Full-time (more than 40 h/week)	10(19)
Stay-at-home parent	8(15)
Student	2(4)
Unemployed	11(21)
Disabled	4(8)
Farmworker	
Yes	19(36)
No	34(64)
Have you felt affected by coronavirus?	
Yes	44(83)
No	8(15)
No Response	1(2)

^*^A total of 55 people participated in one of the focus groups; only 53 completed the socio-demographic survey

As seen in Fig. 1, the intersection of structural factors and SDOH shaped perceptions around the coronavirus and COVID-19 influencing attitudes and behaviors related to the use of COVID-19 testing services and vaccine intentions. Themes of misinformation, shaped by structural factors emerging from historically based distrust in government, public health, and medicine, as well as social factors of insecurity and fear linked to present day employment and deportation concerns emerged in participants’ discussions across focus groups. These intersection of structural and SDOH linked to historically based inequalities place this Latinx and Indigenous Mexican immigrant population in vulnerable positions. These positions impose on them multiple forms of exclusion, discrimination, and violence that shape attitudes about the coronavirus and its spread (e.g., government attempt to exterminate the Latinx population) and behaviors regarding decisions about COVID-19 testing and vaccination, which ultimately increase their risk for COVID-19 exposure.

### Misinformation

Misinformation, i.e., incorrect or misleading information, contributes to confusion, skepticism, and/or disbelief in the COVID-19 virus and vaccination. Information about COVID-19 comes from a myriad of sources that vary from individual to individual. Participants noted some members of their communities might not have access to the internet, proficient English language skills, or knowledge about how to access reliable public health sources for information regarding COVID-19. Instead, many rely on word of mouth or social media platforms like Facebook and Snapchat. One Latinx focus group member said: “I think there should be more groups that help neighbors who do not know very well how to use the internet.” Whereas others expressed frustration with fellow community members who did utilize the internet. A Latinx participant commented: “But if they have Facebook, if they have Snapchat, I do not understand, what is it. If they are up to date in that aspect, they must also be a little bit more up to date about the [COVID-19] tests that are offered, right?”.

Lacking reliable and trustworthy information sources while having access to misinformation was common. Sources of misinformation contributed to the commonly held belief that people would get infected by going to testing sites. A female Latinx participant said: “A lot of people say they’re not going to get tested because they’re going to get contaminated there.” Another Latinx participant shared, “people say that by getting tested you will get the virus.” In addition, misinformation increased the fear for possible secondary effects of the COVID-19 vaccine:

There is a lot of people that are fearful about secondary effects [of COVID-19]. They think that it has several effects. We also think that, perhaps, instead of getting cured, you will get sicker, more badly, because maybe you have the virus . . .

A major source of misinformation was the fragmented and disunited response from the previous government. A Latinx participant commented:

Perhaps they [community members] are confused because they see the Donald Trump administration, and he said, ‘No, no, no.’ . . . I think people are confused about believing whether the virus is serious or not.

This lack of certainty and acknowledgement of the severity of the virus on a national level contributed to some community members not taking COVID-19 seriously. This was evident with people continuing to partake in behaviors that increase risk of spread of the virus. As will be expanded upon below, the misinformation and confusion about COVID-19 undermined trust in public institutions. Mistrust is a byproduct of the misinformation and confusion around COVID-19: community members do not know what is true or what is false. A Purépecha participant made a plea for “public health experts and doctors to bring information to communities, that is correct, so that people are no longer confused…”.

### Insecurity

Insecurity around employment and residency (e.g., fear of deportation linked to undocumented status) also shaped decision making around COVID-19 testing. Many participants shared fear within their community about accessing COVID-testing services due to the possibility that a positive test result could lead to loss of employment or deportation: “There is fear that you can lose your job if you test positive for the virus.” Migration and documentation status makes it difficult for people to get tested. “They fear that they [testing service] will share their [personal] information.” Additionally, misinformation and mistrust in institutions have led to increased levels of insecurity and fear among participants in our focus groups. There is fear of the government locating this immigrant population and going after them. One participant shared the following about a friend: “He’s afraid because they’re saying the government wants to wipe out the whole town."

Immigration and citizenship status are SDOH that create insecurity and barriers to get COVID-19 testing services. Status also shaped ideas around anticipated vaccination. Identification and being identified as undocumented are significant concerns among Latinx farm-working communities. Participants stated that the first thing requested at COVID-19 testing centers is a form of official identification. A Latinx participant commented: “Many people who live in [community names], they have no ID. So many people don’t get the test because they don’t have documents, and they don’t have identification."

### Distrust: Lack of trust in institutions

Lack of confidence in government entities (e.g., the political administration, public health), due to the anti-immigrant political context, played a major role in the attitudes and beliefs held by community members. Participants talked about community perceptions of the government and public health working together to harm minority groups. One Latinx participant commented: “In the beginning, really early on, when the virus was just starting, I was hearing everywhere, they said it [the spread of the virus] was political.”

Across focus groups, participants discussed the role of government in the pandemic. Some discussed community members’ fear that the government incited the virus as a form of state control. A female Latinx participant mentioned: “I heard that if you get the vaccine, it’s so the government can control you.” Others shared concerns that the vaccine is harmful. A Purépecha participants commented: “If we get vaccinated, we might get sicker or are going to die.” Additional beliefs or “myths,” as participants referred to them, about the vaccine included: the COVID-19 vaccine was actually a way for the government to implant a microchip to control and monitor behavior, or it contained another deadly virus. A female Latinx participant illustrated this point: “My neighbors say ‘No’ [to getting the vaccine], because they [the government] are going to put a chip in them, or because they might put another virus [in them]… that’s what people from my community think.”

In light of the mistrust in government, participants vocalized wanting governmental leaders to get vaccinated first so they could observe the vaccine’s effects. A female Latinx participant shared: “When the vaccine comes out [and] when government officials get vaccinated…let’s look at their reaction.” Participants did not want to be test subjects or “guinea pigs” for the vaccine, they feared a repeat of the harm caused by previous medical studies on minorities.

Lack of trust in institutions extends beyond government into hospitals and across the healthcare system. A female Purépecha participant said: “I have heard that people don’t want to go to the hospital because they let you die.”

## Discussion

The current body of literature on the impact of COVID-19 on vulnerable populations is limited in scope, particularly in terms of studies involving Black, Indigenous, and People of Color (BIPOC), including Latinx and Indigenous Latin American immigrant communities, as well as studies providing in-depth qualitative insights about the structural and social factors that shape understanding of the virus, it’s spread, and use of COVID-19 related healthcare services. Our study deepens understanding of the intersection of structural and SDOH as they play out in the COVID-19 pandemic among vulnerable immigrant communities and establishes a baseline data understanding of the role of misinformation, insecurity around employment and deportation, and lack of trust in institutions in shaping attitudes and behaviors around COVID-19 testing and vaccination.

Our findings provide evidence of how a history of institutionalized racism, classism, and colonialism (e.g., evidenced by English-dominant healthcare and public health systems) act as a structural determinants of health and present-day anti-immigrant sentiments as SDOH, which intersect to shape understanding of the coronavirus and COVID-19 and ultimately risk for contagion and disease. Within these immigrant communities, there is a persistent fear that vaccination for COVID-19 is intended to control the Latinx and Indigenous Latin American population in the US. As our findings highlight, community members fear that COVID-19 vaccination will result in implantation of a microchip tracking device, genocide (the deliberate and systematic destruction of their community), and/or COVID-19 contagion. Such findings speak to a community-based lack of trust in government and public health systems connected to historically based abuses and discriminatory practices.

Marginalized and vulnerable populations have valid reasons to distrust the government [48]. The history of medical experimentation on racial and ethnic minorities in the US and abroad[48] has contributed, over centuries, to mistrust and suspicion of institutions associated with authority and the production of medical knowledge, including the academic/scientific community, public health and healthcare systems, and government. The mistreatment from government inform the strong sentiment among community members that the “government wants to end the whole town.”

Add into this dark and oppressive history, the anti-immigration and xenophobic attitudes promulgated by policies of exclusion, such as the public charge enacted by the former Presidential Trump’s administration, which negatively affect immigrant health [49, 50]. Fear and apprehension to trust governing bodies shape ideas around COVID-19 testing and vaccination; to get tested or vaccinated means trusting in healthcare and public health agencies. Rebuilding trust in institutions within vulnerable populations is critical to addressing structural and SDOH in an effort to reduce barriers to healthcare services use, specifically after the tremendous health disparities exposed during the COVID-19 pandemic, that led to disproportionate rates of COVID-19 among communities of color in the US.

One way to (re)build trust in institutions is to engage those most vulnerable to COVID-19 (i.e., BIPOC) in decision-making around public health outreach and service delivery. Negative attitudes about the COVID-19 vaccine among health care workers can decrease vaccine acceptance more broadly, whereas positive interactions with COVID-19 vaccination, such as seeing local health care workers advocate for the vaccine increase vaccine acceptance. Our findings echo this point, positive COVID-19 messages from providers and trusted members of the community such as community health workers rather than government and healthcare or public health institutions increases vaccine acceptance [51]. For instance, recent research found support for COVID-19 vaccines by the Centers for Disease Control and World Health Organization were more influential in vaccine acceptance and uptake than support by government leaders such as former Presidents Trump and Biden [52]. As our study and others find, a lack of trust in governments, especially among marginalized populations, influences decisions around COVID-19 testing and vaccination [53]. Promotion of COVID-19 testing and vaccination by medical leaders and trusted community members such as *promotores de salud* can positively shape COVID-19 decisions and potentially reduce anxieties linked to past and current abuses and discrimination and increase testing utilization and vaccine acceptance and uptake [54].

Additionally, attention should be focused on delivering public health information and news in ways that are accessible to diverse communities, particularly underserved and marginalized communities who may not have access to mainstream media sources, English proficiency, or broadband Internet connection. Our research encourages sharing of COVID-19 material for vulnerable Latinx communities through community and ethnic media sources such as print, radio, and television. Other approaches may include *plaáticas* talks led by *promotores* or other trusted members of the community such as religious leaders (e.g., priests) or medical doctors well-integrated into the community.

Limited access to community evidence-based knowledge sources can perpetuate misinformation. During discussions about community perceptions in our study, one persistent perception arose, hospitals discriminate against Latinx immigrant communities and “let people die.” Throughout the pandemic, hospitals across the nation have been short on beds, ventilators, staff, and other medical resources. A major goal of public health safety measures has been to slow the spread of the coronavirus to flatten the curve, preventing surges in infection and the number of infected patients, thereby reducing the burden on healthcare systems, staff, and medical resources. [55] Yet, as research shows, the key to flattening the curve involves effective social distancing in the community and face masking/covering efforts [56].

As our findings indicate, throughout the pandemic, including in the spike of virus spread in fall 2020 and winter 2021 in the US, community members in the ECV continued to gather for celebrations and did not adhere to strict social distancing measures. Ideas such as those shared by participants in our study, could contribute to lack of trust in health care systems and public health. This finding suggests the need for stronger efforts to share information on the purpose of social distancing, what it means to flatten the curve, and what happens when severe cases of COVID-19 enter care (i.e., healthcare systems). For instance, information about common treatments for severe cases of COVID-19, as well as survival rates for intubated patients, could be widely disseminated to reduce fear and build trust in medicine and public health. Such efforts could dispel misinformation and myths around the virus and its spread and deepen understanding of the meaning and purpose of COVID-19 public health safety measures.

### Limitations

Study findings offer insight into the factors shaping COVID-19 testing and vaccine hesitancy among structurally vulnerable Latinx communities. However, several limitations should be considered when interpreting the findings. First, we conducted this research in August 2020 to January 2021, with focus group data collection in November and December 2020, prior to the emergency approval of COVID-19 vaccines. Thus, discussions about vaccine hesitancy were centered on ideas about future COVID-19 vaccines and the possibility of vaccination, not actual COVID-19 vaccines and vaccination (accessible in 2021 post data collection). Second, we used a snowball sampling technique and recruited participants via *promotores’* social networks within Latinx and Indigenous Mexican immigrant, farm-working communities. Participant experiences may not reflect those of Latinx and Indigenous Latin American immigrants in farm-working communities in other regions or non-immigrant communities. Last, our team conducted focus groups in Spanish and Purépecha and excluded English-speaking Latinos and other indigenous-language speakers. Thus, our findings may not reflect the experiences of more acculturated and formally educated, US born/non-immigrant, and/or English-speaking Latinx community members and other Indigenous Latin Americans.

Despite these limitations, our initial work informed several subsequent steps. First, our network of *promotoras de salud* and investigative team was invited to be part of a larger health equity collaborative in the Coachella Valley and continued to support COVID-19 testing and then vaccination in the valley post study period. Second, our capacity building and community driven research approach informed a larger study funded by the National Institute of Health (NIH) Community Engagement Alliance (CEAL) Against COVID-19 Disparities (https://covid19community.nih.gov/). This study, part of a state-wide alliance, Share, Trust, Organize, Partner COVID-19 California Alliance (STOP COVID-19 CA) focused on health equity in COVID-19, is ongoing and engages African American/Black, Latinx/Indigenous Latin American, and Indigenous/Native American communities in addressing COVID-19 related disparities in the Inland Southern California region. The model to build capacity of community leaders (i.e., *promotores de salud*) and students in collecting and analyzing data discussed in this article was replicated and used in the subsequent research [57]. The findings have been disseminated widely in the ECV through social media and community talks that continue to inform engagement of vulnerable immigrant communities [58]. The findings unique to Latinx/Indigenous Latin American communities have also been disseminated in academic and public health communities in the US and Mexico via a recent publication in CONAPO: Consejo Nacional de Población sponsored by the Mexican government [59].

## Conclusions

Structural and social factors increase risk for COVID-19 transmission and death. Mistrust in the government and fear of deportation due to anti-immigrant sentiments and policies may create significant barriers to utilization of COVID-19 testing services and vaccine uptake among Latinx immigrant communities across the US. Solutions must focus on addressing the structural and SDOH (including racism, classism, and discrimination) that exacerbate the health disparities experienced among vulnerable populations in the context of COVID-19. This study advocates for the need to acknowledge the role of structural inequities in Latinx and Indigenous Latin American immigrant communities to further foster trust with institutions (government, public health). This acknowledgment can help to increase confidence and trust in COVID-19 testing and vaccination services among Latinx and Indigenous Latin American immigrant communities in the US.

## Data Availability

The datasets used and/or analyzed during the current study are available from the corresponding author upon reasonable request.

## Abbreviations

BIPOC: Black, Indigenous, and People of Color
CBPR: Community based participatory research
COVID-19: Coronavirus disease 2019
ECV: Eastern Coachella Valley
SARS-CoV-2: Severe acute respiratory syndrome coronavirus 2
SDOH: Social determinants of health
